# Maternal and child FUT2 and FUT3 status demonstrate relationship with gut health, body composition and growth of children in Bangladesh

**DOI:** 10.1038/s41598-022-23616-9

**Published:** 2022-11-05

**Authors:** Md. Amran Gazi, Shah Mohammad Fahim, Md. Mehedi Hasan, Farzana Hossaini, Md. Ashraful Alam, Md. Shabab Hossain, Md. Daluwar Hossain, Subhasish Das, Rashidul Haque, Mustafa Mahfuz, Tahmeed Ahmed

**Affiliations:** 1 Nutrition and Clinical Services Division, International Centre for Diarrhoeal Disease Research, Bangladesh (Icddr,B), Mohakhali, Dhaka, 1212 Bangladesh; 2 Office of the Executive Director, International Centre for Diarrhoeal Disease Research, Bangladesh (Icddr,B), Dhaka, 1212 Bangladesh; 3 Department of Global Health, University of Washington, Seattle, WA USA; 4 Department of Public Health Nutrition, James P Grant School of Public Health, BRAC University, Dhaka, 1212 Bangladesh; 5 Infectious Diseases Division, International Centre for Diarrheal Disease Research, Bangladesh (Icddr,B), Dhaka, Bangladesh

**Keywords:** Genetics, Biomarkers, Gastroenterology

## Abstract

Fucosyltransferase 2 (FUT2) and 3 (FUT3) may influence host biological functions. We aim to assess the relationship between maternal and child FUT2 (Secretor) and FUT3 (Lewis) status with growth, body composition, gut health and histologic features in Bangladeshi children. We conducted a case–control study where secretor and Lewis status were ascertained from saliva samples of 408 mother–child dyads. Upper-arm fat area estimate (UFE) and total upper arm area (TUA) were found higher among children of Lewis negative mothers (*p* = 0.01 and *p* = 0.07, respectively). Changes in UFE after nutrition intervention were significantly greater among Lewis positive children than those of negative for Lewis (*p* = 0.05). Significant differences were observed for child UFE based on secretor and Lewis status of the mothers (*p* = 0.04). Lewis positive children had greater changes in WAZ (*p* = 0.07) and WLZ (*p* = 0.02) than Lewis negative children at the end of nutrition intervention. Fecal Reg1B was elevated in secretor positive children compared to their counterparts (*p* = 0.03). Lewis negative children had higher concentrations of MPO compared to Lewis positive children (*p* = 0.08). We also observed a higher frequency of subtotal villous atrophy among secretor negative and Lewis positive children (*p* = 0.09 and *p* = 0.01, respectively) than those of their counterparts. The findings provide insights for further studies to elucidate causal influences.

## Introduction

The early years of life are crucial for ample physical, socioemotional, cognitive and motor development^1^. A spectrum of metabolic, immunological as well as physiological adaptations during this period is known to modify child growth, body composition and subsequent disease risk. Illness and exposures to infectious agents curb the optimum growth potential in young children^2^. Inappropriate growth and sub-optimal nutritional status may also affect the body fat distribution as well as muscle mass during the critical period of growth and development^3^. Body composition at younger age may also influence the disease risk at later life^4^. Prior works suggest that tissues such as muscle, fat and bone are closely associated with the regulation of whole-body energy-metabolism and can be regulated by genetic factors^4^. However, evidence on relationship between genetic factors, specifically on the secretor and Lewis status with childhood growth, body composition, gut health and histologic features are scarce.

Histo blood group antigens (HBGAs) containing ABO, fucosyltransferase 2 (FUT2, Secretor gene) and 3 (FUT3, Lewis gene) act as host genetic determinants that may differentially impact susceptibility of people and their children to enteric infections^5^. HBGAs are synthesised through sequential additions of monosaccharides to disaccharide precursors with the catalyzation of various glycosyltransferases. Three major HBGA gene families are involved in the catalization namely secretor, Lewis and ABO families that code an α-1,2 fucosyltransferase (FUT2), an α-1,3 or α-1,4 fucosyltransferase (FUT3) and two glycosyltransferases (A and B enzymes), respectively^6^. Previous studies found the association of human milk oligosaccharides (HMO) composition with child growth and body composition^7,8^. Combinations of FUT2 and FUT3 phenotype in breastfeeding mothers influence the concentration, distribution and types of HMOs expressed in breast milk. A 2015 study found that, infants of non-secretor mothers are delayed in the formation of a bifidobacteria containing microbiota may be due to fact that infant were not able to consume the specific HMOs delivered by the mother^9^. Gut microbiota composition affects our ability to metabolize nutrients, and to regulate immune function and defend against a multitude of pathogens^10^. The HBGA genes are responsible for the synthesis of antigens expressed on intestinal mucosa and deposited into digestive secretions^6^. Considering its significance, it is essential to understand the relationship between HBGAs gene function with regards to intestinal health and histological features in children.

Overall, there is a complex relationship and there remains paucity of data on maternal and child HBGAs status and gut health, body composition and growth of children, particularly in those who are living in resource limited settings. Moreover, the prevalence of FUT2 and FUT3 enzymes activity varies strongly depending on geography within the population^11^. To fill this gap, we aim to assess the maternal and child FUT2 (Secretor) and FUT3 (Lewis) status on the body composition, growth, gut health and histologic findings of Bangladeshi children.

## Materials and methods

### Study design, settings, population and sample size

In this analysis, we have included 408 children from the Bangladesh Environmental Enteric Dysfunction (BEED) study. The BEED study was a community-based nutrition trial done in a slum settlement in Dhaka, Bangladesh. The detailed methodology of the BEED study has already been published elsewhere^12^. In brief, children aged 12–18 months and having a LAZ <  − 1 were enrolled in the study. They have received nutrition intervention (daily an egg, 150 ml of milk, and micronutrient sprinkles) over a period of 90 feeding days. At the end of intervention, the children were categorized into two groups: children with an improvement in the length-for-age Z score (LAZ) after the nutrition intervention and children who failed to improve their LAZ after the supplementation. A total of 120 children who failed to improve their LAZ after the nutritional therapy underwent upper GI endoscopy after obtaining proper clinical history from the parents and screening for any other diseases, for instance tuberculosis, celiac diseases and other causes which might have resulted in malnutrition. The current analysis was a case–control study where children with an improvement in the length-for-age Z score (LAZ) after the nutrition intervention was the cases and children who failed to improve in their LAZ after the nutrition intervention was the controls. Based on previous literature and considering the level of significance as 0.05 and 90% power, the sample size was calculated as 410 (205 cases and 205 controls)^13,14^. Among the children enrolled in the BEED study, saliva samples were available from 408 children who were included in this present study. We also included the mothers of the same children. Therefore, a total of 816 mother–child dyads (408 children and 408 mothers) were included in this analysis. Among the endoscopy done children, we were able collect the saliva samples from 85 children and were available for histological features analysis. All the information was collected at enrolment and after the completion of 90-day nutrition intervention.

### Ethical procedure

An Institutional Review Board (IRB) of icddr,b, Mohakhali, Dhaka, Bangladesh has approved the research protocol and all methods were carried out in accordance with relevant guidelines and regulations. Parents/caregivers of the children and mothers were informed about the study and informed written consent was obtained at enrolment as well as prior to upper GI endoscopy of the selected children.

### Data and specimen collection, processing and storage

Socio-economic status information was obtained from the caregivers of the children by trained field staff at enrollment. Weights and lengths of the participants were measured before and after the nutrition intervention using Seca model 345 and Seca model 417 respectively (Seca GmbH & Co. KG., Hamburg, Germany). WHO anthropometry calculator was used to measure the indicators of nutritional status (LAZ, WAZ, WLZ). Body composition measures including total upper arm area (TUA), upper arm muscle area estimate (UME) and upper arm fat area estimate (UFE), were calculated following the formulas from published literature^15^.

Saliva samples were collected from mother and child pairs using Oracol saliva collecting device (S10, Malvern Medical Developments Ltd., Worcester, United Kingdom) after enrolment. The samplers were handled like toothbrushes and rubbed the sponge along the gum line for 1 min. Mother had collected the saliva samples herself while trained field staff had collected the saliva samples from the children following standard operating procedures. The samplers were then reinserted into the Oracol tubes, sponge down and quickly transported from the field site to the laboratory at icddr,b, maintaining a cold chain. Samples were centrifuged sponge up for 10 min at 1,500 g and then centrifuged again for 5 min at 10,000 g; the supernatant was transferred into a separate vial. Stool samples were collected to assess the role of secretor and Lewis status on gut health biomarkers namely Myeloperoxidase (MPO), Neopterin (NEO), Alpha-1 anti-trypsin (AAT) and regenerating gene 1B (Reg1B). Aliquots of saliva and stool were immediately frozen at −80 °C, pending analysis.

### Determination of secretor status, Lewis type and ABO phenotyping

The ABO histo-blood group phenotype of secretor-positive individuals and the Lewis phenotype ((Lewis a (Lea) and Lewis b (Leb)) were determined using a saliva-based enzyme-linked immunosorbent assay (ELISA), essentially as described and used previously by our group^14^. Optical Density (O.D.) at 450 nm was measured using an ELISA reader. A cut-off value of 0.10 or greater was deemed as positive for all the antigens. From this data, secretor and Lewis status and also the blood group (A, B and O) of secretor positive individuals were determined. Individuals were considered as secretor-positive if Leb, and/or A and/or B were obtained in saliva. Participants were defined as Lewis-positive if either Lea or Leb antigen was observed. Consequently, a child was regarded secretor-negative if Lea was detected, and Lewis-negative if neither Lea nor Leb were present in saliva. However, for a number (n = 32) of Lewis-negative individuals, results from the HBGA assay was inadequate to confirm secretor phenotype. Thus, secretor status was further ascertained by UEA-1 lectin assay described before^16^.

### Analyses of gut health biomarkers by ELISA

Stool samples were assayed for NEO (GenWay, San Diego, California), MPO (Alpco, Salem, New Hampshire), AAT (Biovendor, Chandler, North Carolina) and Reg1B (TechLab, Blacksburg, Virginia) using commercially available ELISAs according to kit manuals. Standards provided by the manufacturers were used to calculate the concentrations of each of the biomarkers.

### Upper gastro-intestinal endoscopy and histopathology

A sub-analysis was conducted that includes children who underwent upper gastro-intestinal endoscopy in the BEED study and given their saliva for this current analysis (n = 85). Biopsies were obtained from the second part of duodenum and fixed in paraffin-embedded 10% buffered formalin. Paraffin sections were then stained by Hematoxylin and eosin/H&E and an expert histopathologist examined for histopathology. The villous height, crypt depth, intraepithelial lymphocytes (IELs) and presence of inflammatory infiltrates in the lamina propria were determined from the biopsied specimens. Abnormalities in intestinal histological characteristics (villous atrophy, crypt hyperplasia, inflammatory infiltrates in lamina propria and IELs) were defined according to the literature published elsewhere^17^.

### Statistical analyses

Socio-economic characteristics and demographic factors were described using frequencies with proportions when variables were categorical. Means and standard deviations (SD) were reported for symmetrically distributed quantitative variables. Medians with interquartile ranges (IQRs) were used when quantitative variables were asymmetrically distributed. To analyze the differences between groups, t-test or Mann–Whitney test was performed for numeric data, and Pearson’s chi-square test or fisher’s exact test was applied for categorical variables. Analysis were also done without considering the outlier values in order to investigate whether removal of outliers can modify the results. Group-wise comparisons of the quantitative asymmetric variables were done using the Kruskal–Wallis test and post hoc analysis was performed to locate specific differences. Delta changes in different body composition parameters (DTUA, DUME, and DUFE) and growth indicators (DLAZ, DWAZ, DWLZ) were calculated by subtracting the endline to baseline data. Statistical significance (*p* < 0.05) and marginal significance (between 0.05 and 0.10) levels were adopted in the current analyses. R version 3.5.1 was used for all the statistical analyses performed (https://www.r-project.org).

## Results

### Sociodemographic and general characteristics of the mother and child

Secretor and Lewis status and the antigen phenotypes of the participants are depicted in Table 1. Prevalence of secretors among children and mothers were 68.7% and 70.8%, respectively; while Lewis positivity was 88.2% and 84.6%, respectively. Considering secretor and Lewis status together, the percentage of both positive children were 60.8%, while it was 59.3% for mothers. The baseline sociodemographic characteristics of the children including nutritional status are presented in Table 2.

**Table 1:** Summary of secretor status and Lewis antigen phenotypes both in mother and children.

Phenotype	Total (child, N = 408)	Total (mother, N = 408)
**Secretor (%)**		
Positive	280 (68.7)	289 (70.8)
Negative	128 (31.3)	119 (29.2)
**Lewis (%)**		
Positive	360 (88.2)	345 (84.6)
Negative	48 (11.8)	63 (15.4)
**Combined (%)**		
Secretor positive/ Lewis positive (Lea–b + or Lea + b +)	248 (60.8)	242 (59.3)
Secretor positive/ Lewis negative (Lea–b–)	32 (7.8)	47 (11.5)
Secretor negative/Lewis positive (Lea + b–)	112 (27.5)	103 (25.2)
Secretor negative/Lewis negative (Lea–b–)	16 (3.9)	16 (3.9)

**Table 2:** Descriptive characteristics of the participants based on FUT (Secretor and Lewis) status of the Children at enrolment.

	Both negative children (N = 16)	Secretor positive Lewis negative children (N = 32)	Secretor negative Lewis positive children (N = 112)	Both positive children (N = 248)	Overall (N = 408)
**Age in days**					
Age in days, Mean (SD)	462 (62.9)	454 (69.4)	457 (64.8)	452 (63.1)	454 (63.9)
**Gender**					
Female sex, n (%)	5 (31.3%)	14 (43.8%)	58 (51.8%)	109 (44.0%)	186 (45.6%)
Male	11 (68.8%)	18 (56.3%)	54 (48.2%)	139 (56.0%)	222 (54.4%)
**LAZ**					
Mean (SD)	−2.08 (0.79)	−2.19 (0.74)	−2.10 (0.76)	−2.14 (0.79)	−2.13 (0.78)
**WAZ**					
Mean (SD)	−1.60 (1.09)	−1.60 (0.80)	−1.57 (0.81)	−1.65 (0.90)	−1.62 (0.87)
**WLZ**					
Mean (SD)	−0.84 (1.14)	−0.73 (0.69)	−0.76 (0.88)	−0.84 (0.94)	−0.81 (0.91)
**MUAC**					
Mean (SD)	14.1 (0.985)	13.8 (0.892)	13.8 (0.917)	13.8 (0.930)	13.8 (0.924)
**Source of drinking water**					
Not improved	0 (0%)	0 (0%)	0 (0%)	1 (0.403%)	1 (0.245%)
Improved	16 (100%)	32 (100%)	112 (100%)	247 (99.6%)	407 (99.8%)
**Water treatment**					
No	5 (31.3%)	10 (31.3%)	59 (52.7%)	101 (40.7%)	175 (42.9%)
Yes	11 (68.8%)	22 (68.8%)	53 (47.3%)	147 (59.3%)	233 (57.1%)
**Sanitation**					
Not improved	7 (43.8%)	10 (31.3%)	45 (40.2%)	88 (35.5%)	150 (36.8%)
Improved	9 (56.3%)	22 (68.8%)	67 (59.8%)	160 (64.5%)	258 (63.2%)
**Wash hands with soap before preparing food**					
Never	5 (31.3%)	9 (28.1%)	51 (45.5%)	91 (36.7%)	156 (38.2%)
Rarely	1 (6.25%)	8 (25.0%)	12 (10.7%)	28 (11.3%)	49 (12.0%)
Sometimes	8 (50.0%)	12 (37.5%)	30 (26.8%)	105 (42.3%)	155 (38.0%)
Always	2 (12.5%)	3 (9.38%)	19 (17.0%)	24 (9.68%)	48 (11.8%)
**Wash hands with soap after using toilet**					
Never	0 (0%)	1 (3.13%)	2 (1.79%)	14 (5.65%)	17 (4.17%)
Rarely	1 (6.25%)	1 (3.13%)	1 (0.893%)	2 (0.806%)	5 (1.23%)
Sometimes	3 (18.8%)	5 (15.6%)	28 (25.0%)	48 (19.4%)	84 (20.6%)
Always	12 (75.0%)	25 (78.1%)	81 (72.3%)	184 (74.2%)	302 (74.0%)
**Wash hands with soap after helping the child to defecate**					
Never	0 (0%)	2 (6.25%)	2 (1.79%)	8 (3.23%)	12 (2.94%)
Rarely	3 (18.8%)	0 (0%)	4 (3.57%)	8 (3.23%)	15 (3.68%)
Sometimes	3 (18.8%)	6 (18.8%)	31 (27.7%)	64 (25.8%)	104 (25.5%)
Always	10 (62.5%)	24 (75.0%)	75 (67.0%)	168 (67.7%)	277 (67.9%)
**Crowding index**					
High (> 4 people sleep in a room)	3 (18.8%)	9 (28.1%)	17 (15.2%)	48 (19.4%)	77 (18.9%)
Low (≤ 4 people sleep in a room)	13 (81.3%)	23 (71.9%)	95 (84.8%)	200 (80.6%)	331 (81.1%)
**Monthly income, USD**					
Mean (SD)	253 (226)	194 (91.4)	190 (98.5)	201 (115)	199 (116)
**Household asset score**					
Mean (SD)	3.63 (2.00)	4.00 (1.57)	3.72 (1.68)	4.02 (1.76)	3.92 (1.73)

### FUT status and body composition

There was a significant difference (*p* = 0.02) in UFE as well as marginal significance in TUA (*p* = 0.07) of children based on Lewis negativity of mother (Figure S2). Children of Lewis negative mother had significantly higher UFE compared to children of Lewis positive mother. Marginal significance was also found for DUFE of children based on child Lewis positivity (*p* = 0.06) which indicate that changes in UFE after nutrition intervention was higher for children with Lewis positive status (Figure S3). We have also categorized the participants FUT status (both children and mother) into four groups based on secretor and Lewis combined and analysed their relationship with body composition: secretor positive/Lewis positive (Le^a–b+^ or Le^a+b+^); secretor positive/Lewis negative (Le^a–b–^); secretor negative/Lewis positive (Le^a+b–^); and secretor negative/Lewis negative (Le^a–b–^). Significant difference was only found for UFE among the children based on FUT status of their mothers using Kruskal − Wallis test (*p* = 0.047). Post-hoc analysis using dunn's test showed that the significant difference was between the children of secretor positive/ Lewis negative mother and secretor positive/ Lewis positive mother (*p* = 0.022) (Figure S5). Other dunn's pairwise comparisons were not statistically significant (Fig. S5). Besides, none other parameters of body composition showed statistically significant results (Figs. S1 and S4).

### FUT status and growth indicators

**Figure 1:**
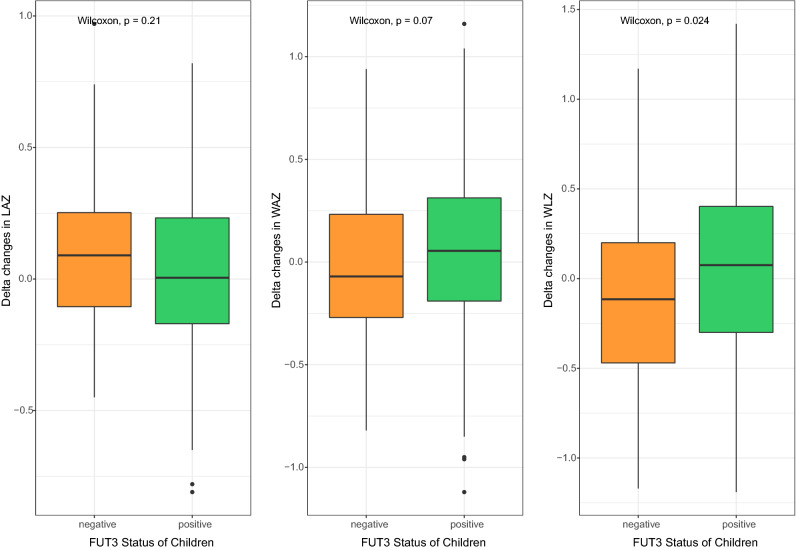
Delta changes in growth parameters in relation to FUT3 status in children (n = 408).

Delta changes in WLZ were significantly higher in Lewis positive compared to Lewis negative children and delta change in WAZ were also marginally significant and higher in Lewis positive children (*p* = 0.02 and 0.07, respectively). However, Delta change in LAZ was not statistically significant between the groups (*p* = 0.21) (Fig. 1). For Lewis positive children, the median (q1, q3) values of DWAZ, DWLZ and DLAZ were 0.05 (−0.19, 0.31), 0.08 (−0.30, 0.40), and 0.005 (−0.17, 0.23) while the values were −0.07 (−0.27, 0.23), −0.12 (−0.47, 0.20) and 0.09 (−0.10, 0.25) for Lewis negative children. Changes in none of the growth parameters were statistically significant on the basis of secretor status of the children; secretor status and Lewis status of the mother. Overall findings pertaining to growth in children are presented in Tables S1 and S2.

### FUT status and gut health

**Figure 2:**
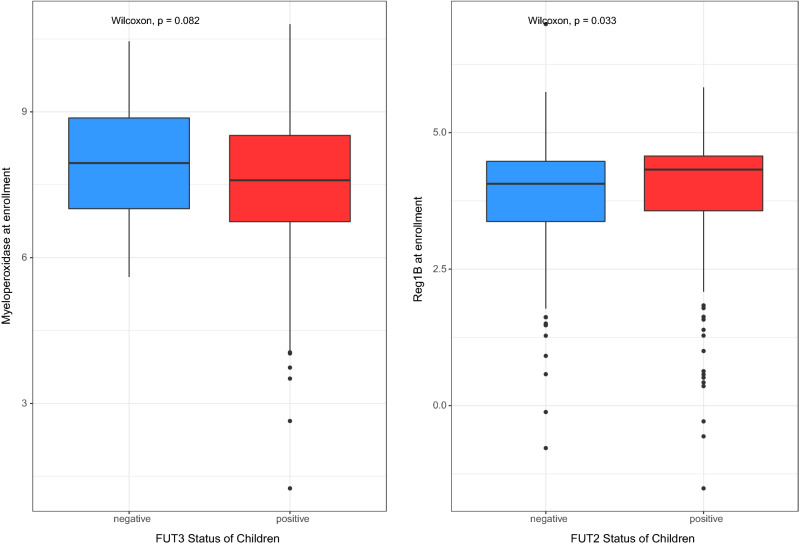
Differences in the levels of MPO and Reg1B in relation to FUT3 status in children (n = 408).

We have analyzed the relationship of secretor and Lewis status with gut health biomarkers (MPO, NEO, AAT and Reg1B) and found that Lewis negative children had higher fecal concentration of MPO at baseline that is marginally significant (*p* = 0.08). On the other hand, secretor positive children had significantly higher fecal concentration of Reg1B at baseline (*p* = 0.03). All other biomarkers did not show the statistically significant results with either secretor or Lewis status of children and mother (Tables S3 and S4). The significant findings are presented in Fig. 2.

### FUT status and histopathological findings

**Figure 3:**
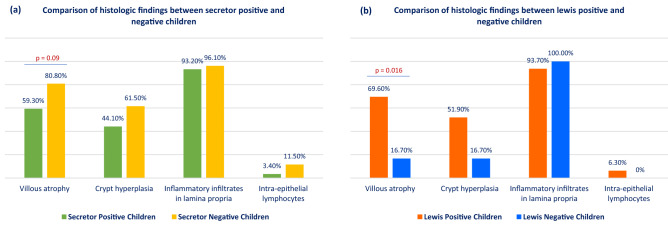
Comparison of histologic findings among children in relation to their secretor and Lewis status (n = 85).

Secretor negative children had higher prevalence of villous atrophy compared to secretor positive children that is marginally significant (80.8% vs 59.3%; *p* = 0.09). However, the prevalence of crypt hyperplasia (61.5% vs 44.1%; *p* = 0.21), inflammatory infiltrates in lamina propia (96.10% vs 93.20%; *p* = 1.00) and IELs (11.50% vs 3.40%; *p* = 0.17) were not statistically significant. On the other hand, Lewis negative children had significantly lower prevalence of villous atrophy compared to Lewis positive children (16.7% vs 69.6%; *p* = 0.02). Prevalence of crypt hyperplasia, inflammatory infiltrates in lamina propia and IELs were not statistically significant considering the Lewis status of the children. The histological findings are presented in Fig. 3.

## Discussion

Our results demonstrated that UFE and TUA were significantly increased in children with Lewis negative mothers. However, delta changes in child UFE after nutrition intervention were significantly increased among children with Lewis positivity. Significant difference was also found for child UFE based on FUT status (both secretor and Lewis) of their mothers and it has been shown that the difference was between the children of secretor positive/Lewis negative mother and secretor positive/Lewis positive mother. Regarding growth parameters, Lewis positive children had significantly higher changes in weight (both WAZ and WLZ) indicating that Lewis negative children are tend to be more underweight and wasted. None of the growth parameters (LAZ, WAZ and WLZ) in children has been changed based on secretor status of the children or secretor and Lewis status of the mother.

HBGAs such as FUT2 (secretor gene) and FUT3 (Lewis gene) may act as inherent host genetic factors that can regulate growth and body composition. Our study participants are stunted and it is obvious that the body's response to malnutrition followed an orchestration in which depletion of body-fats and muscles occur first and retardation of body-composition happen if undernutrition continues^18^. Therefore, the anthropometric measures using upper-arm are very useful to monitor the growth and body-composition, nutritional status and evaluating the outcomes of intervention^18^. Several studies have shown the association of upper-arm composition with diseases, biochemical changes, clinical diagnosis and nutritional status^19–21^. Previous studies indicated that secretor and Lewis status have remarkable effects on HMO and microbiota composition while certain HMOs and microbiota might play a role in changing the body composition and that change eventually takes part in infant growth. In a study by Alderete et al., has shown the significant relations between HMOs and infant body composition where Lacto-N-Fucopentaose I was found to be inversely associated with infant lean mass, fat mass and overall weight^22^. Another study in adults found the correlation of gut flora richness with obesity and metabolic markers, conferring support to the theory that variations in HMOs may alter the gut microbiota, modulate gastrointestinal activities, and regulate inflammatory processes, thereby influencing infant growth and body composition^22^. In a small cohort conducted on high and normal weight gain breastfed infants found significantly different levels of HMO to exclusively breastfed infants with excessive weight gain. The results suggest that certain HMOs, including 2′-Fucosyllactose which is the most abundant HMO could be part of the weight gain. The study also found that some HMOs were associated with anthropometry and body composition^23^.

Overall, secretor and Lewis status exert their impact on HMO and microbiota composition and that may have direct associations with infant growth and body composition either by their effects on epithelial cell responses in the intestine or through systemic effects of HMOs^24^. Previous study reports that milk metabolome of Lewis negative mothers differ in its non-HMO metabolite composition from mothers with other HMO phenotypes^25^. In addition, maternal weight and body composition have recently been associated with growth in infancy, showing that concentrations of lacto-N-hexaose and difucosyllacto-N-tetrose were higher in overweight and obese compared to control mothers^26^. Infants fed by non-secretor mothers are delayed in the establishment of a bifidobacteria due to difficulties in consuming the specific HMOs delivered by the mother^27^. However, there are other factors that could exert their impact on growth and body composition and might have link with secretor and Lewis status. For instance, study conducted in Bangladesh showed that children whose mothers were participated in the women’s group intervention have had a positive impact on their growth and body composition^28^.

We have investigated the relationship of FUT status with gut health biomarkers preferably MPO, NEO, AAT and reg1B. These biomarkers are thought to involved in intestinal inflammation, epithelial damage in intestine, intestinal permeability and microbial translocation^29^. We found that Reg1B levels were found to be significantly higher in secretor positive than the secretor negative children. Reg1B is a marker of epithelial tissue injury and subsequent repair in the small intestine^29^. Diverse bacterial population is one of the most prominent factors in overall gut health and it has been found that non-secretors have less diversity in their microbiomes compared to secretors^30,31^. Moreover, non-secretors have only one fifth of the enzyme, intestinal alkaline phosphatase (IAP), that secretors possess^32^. IAP is an important enzyme in the gut that plays an essential role in intestinal health and maintaining homeostasis. So, it is expected that the Reg1B is lower in secretors which contradicts our finding. Then again, Reg1B is known to have antibacterial effect and expressed in human paneth cells^33^. It is also shown that the secretors are more prone to pathogenic infections and that might be involved with the increased levels of Reg1B through their antibacterial effect. In addition, dietary modifications can create a significant difference in overall gut health for non-secretors. Besides, Lewis negative children had significantly higher concentration of MPO at enrolment than their counterpart. MPO is a marker of neutrophil activity, suggestive of intestinal inflammation^34^. Lewis-negativity was associated with increased susceptibility to shigellosis in a cohort of infants in Bangladesh^35^. Consequently, it was shown that MPO were elevated during Shigella infections that may increase the level of MPO through neutrophil activation and infiltration^36^. In addition, FUT status of mother can affect the distribution and concentration of HMOs expressed in breastmilk. Therefore, changes in breastmilk composition can influence the child’s microbiome, which in turn may alter resistance to enteric infections^14^. However, we did not have the individual pathogenic data to prove all these connections. We also could not able to measure the microbiome diversity and IAP status of our participants. Overall, there is a complex interplay between the FUT status, EED and gut health biomarkers and warrant further exploration.

In this study, we explored the histological features of the intestinal mucosa in a sub-group of malnourished children and compared the differences among the individuals based on their FUT status. The study found that secretor negative and Lewis positive individuals had significantly higher frequency of subtotal villous atrophy than their counterparts. In addition, other histological features followed the same trend for secretor negative and Lewis positive children, except the inflammatory infiltrates in lamina propia which was slightly higher for Lewis negative children. The non-secretor state and Lewis status was found to be significantly associated with celiac disease in earlier study^37^. Celiac disease is the best-known cause of villous atrophy. However, celiac was found to be uncommon among the people in the same region in our earlier analysis^38^. Participants in the current study were slum dwellers and lived in a poor socioeconomic environment where EED and bacterial overgrowth in intestine is common^39–41^. Therefore, conditions like EED and bacterial overgrowth may explain why villous atrophy was higher in secretor negative participants. Besides, the Lewis b and H antigens are receptors for the bacteria *Helicobacter pylori*, a gram-negative bacterium that can cause gastritis and results in protein losing enteropathy^42^. This condition eventually might be associated with higher villous atrophy in Lewis positive children.

The current study has several shortcomings, which are-firstly, the HMO concentrations should have been investigated to find out the potential modifiable factors and their relationship with FUT status and study outcomes. However, breast milk samples from mother were not available in the current analysis. Moreover, the causal effect of secretor and Lewis on the factors listed above remains to be proven in this study. Investigation of a large number of participants to understand the relationship of secretor and Lewis with gut health, growth and body composition is the major strength of the study. However, some p-values were marginal and further large-scale analysis seems to be desirable including control subjects. In addition, multiple testing corrections can be done in the future with appropriate study design. A substantial number of participants were underwent endoscopy and a detail analysis on secretor and Lewis with the histological features is also a unique findings of this study.

## Conclusions

In this study, WAZ and WLZ of children were found to be significantly increased among Lewis positive children indicating that Lewis negative children are tend to be more underweight and wasted. In addition, upper arm fat area was significantly increased among the children of Lewis negative mother and also delta changes in upper arm fat area were significantly increased after nutrition intervention among Lewis positive children. This mean that the positive changes in body composition is higher in Lewis positive children and children of Lewis negative mother. There is also a role of child secretor positivity and Lewis negativity in increasing the gut health biomarkers. In contrast, intestinal villous atrophy was significantly increased among secretor negative and Lewis positive children.

## Supplementary Information

Supplementary Information 1.

Supplementary Information 2.

Supplementary Information 3.

Supplementary Information 4.

Supplementary Information 5.

Supplementary Information 6.

Supplementary Information 7.

Supplementary Information 8.

## Data Availability

All data generated or analysed during this study are included in this published article [and its supplementary information files].
