# Supplementary figures and images for: Maternal and child FUT2 and FUT3 status demonstrate relationship with gut health, body composition and growth of children in Bangladesh
Source: Sci Rep. 2022 Nov 5;12:18764. doi: 10.1038/s41598-022-23616-9 (PMC9637127; doi:10.1038/s41598-022-23616-9)

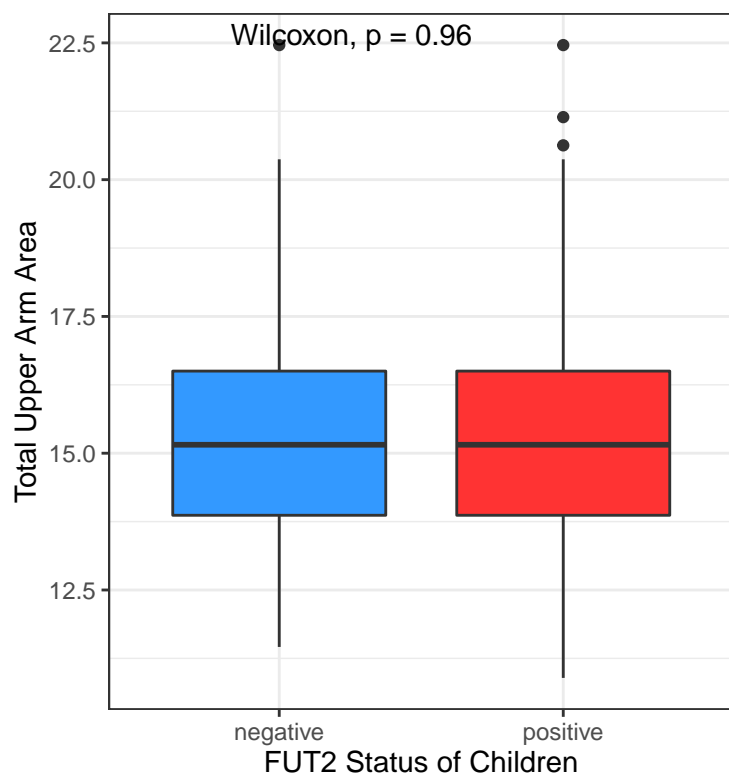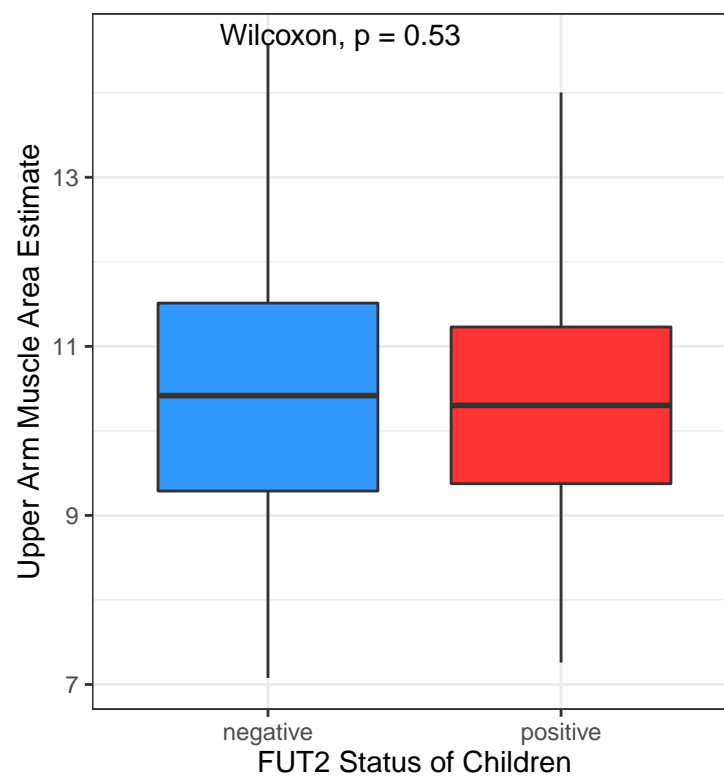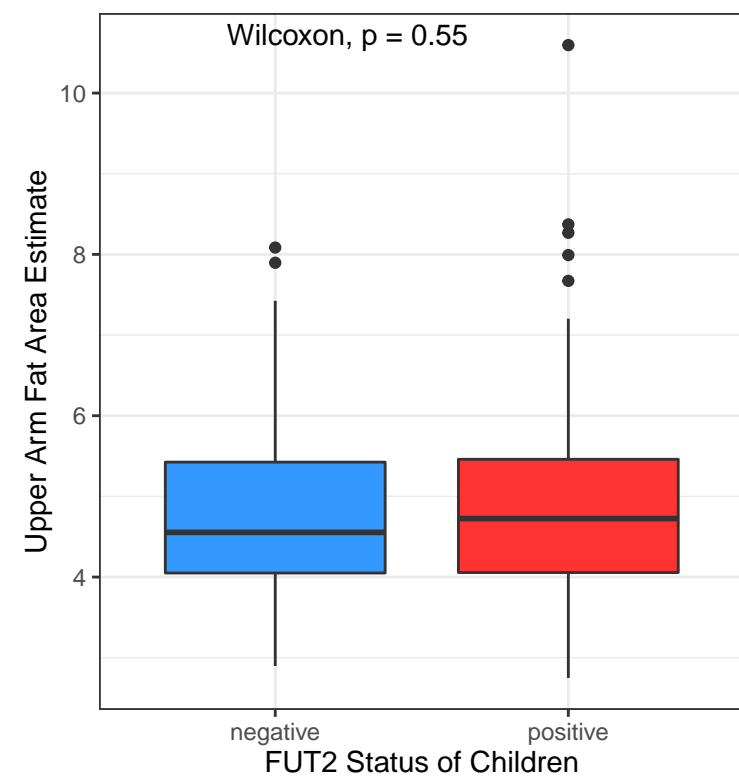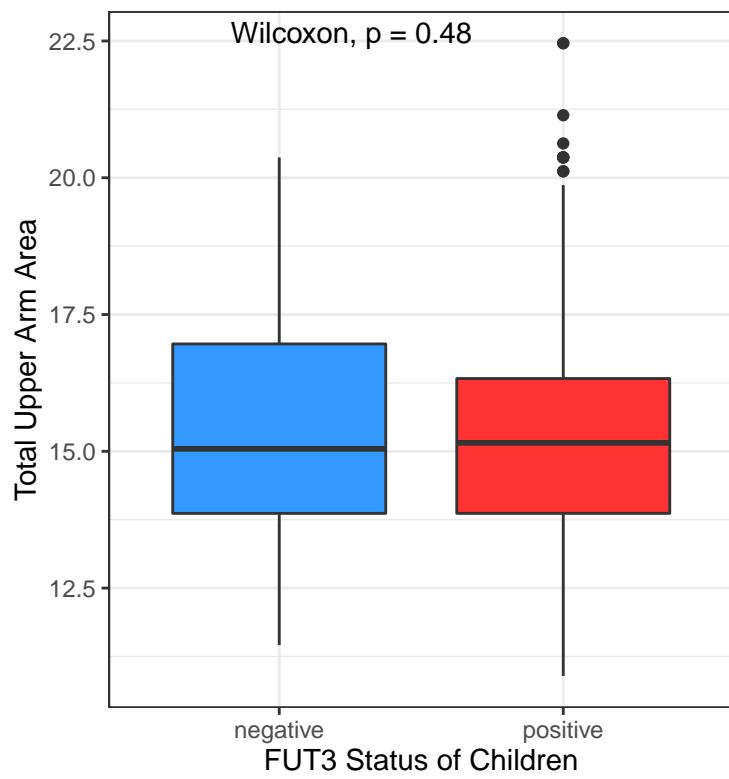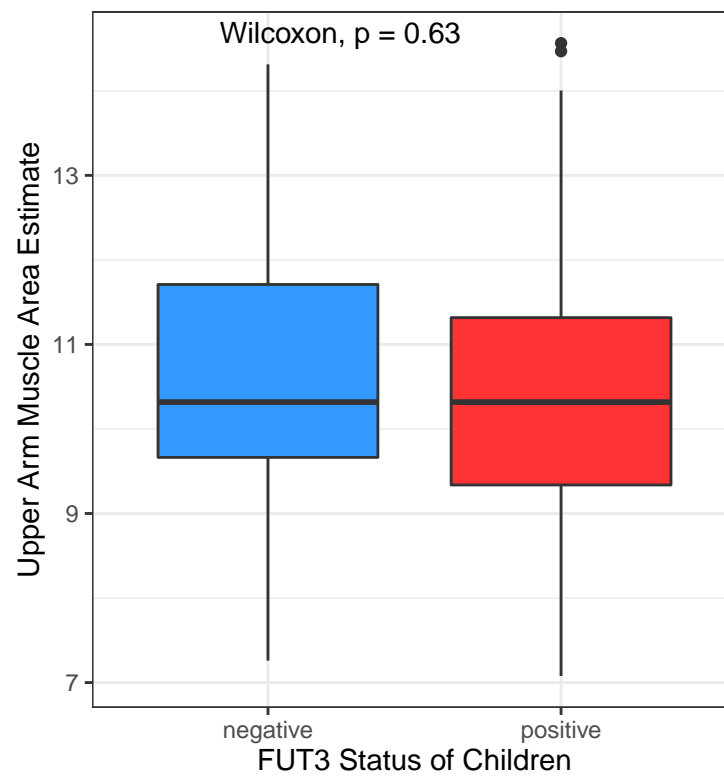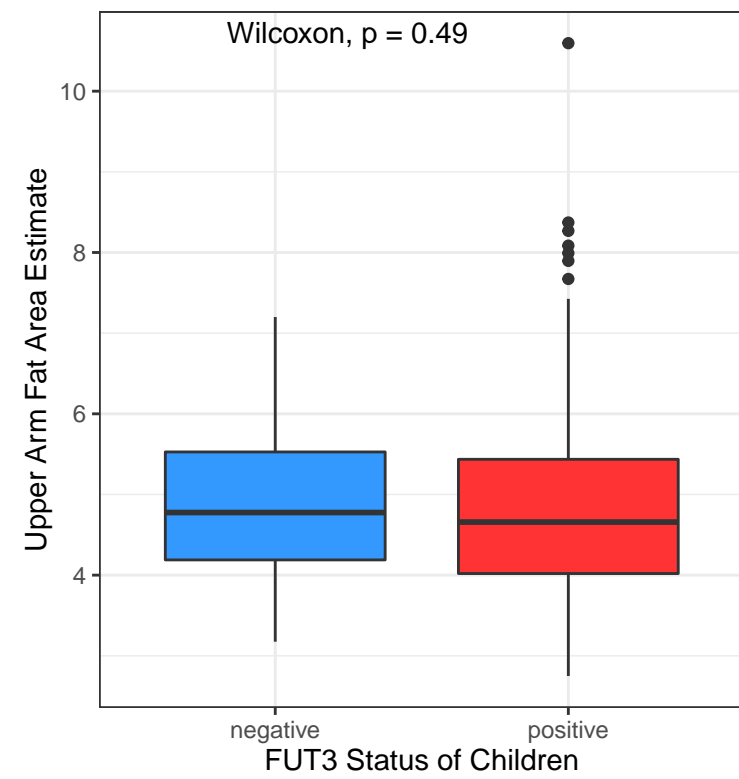

Supplement: Supplementary file 1 — Supplementary Information 1. [file 41598_2022_23616_MOESM1_ESM.pdf]

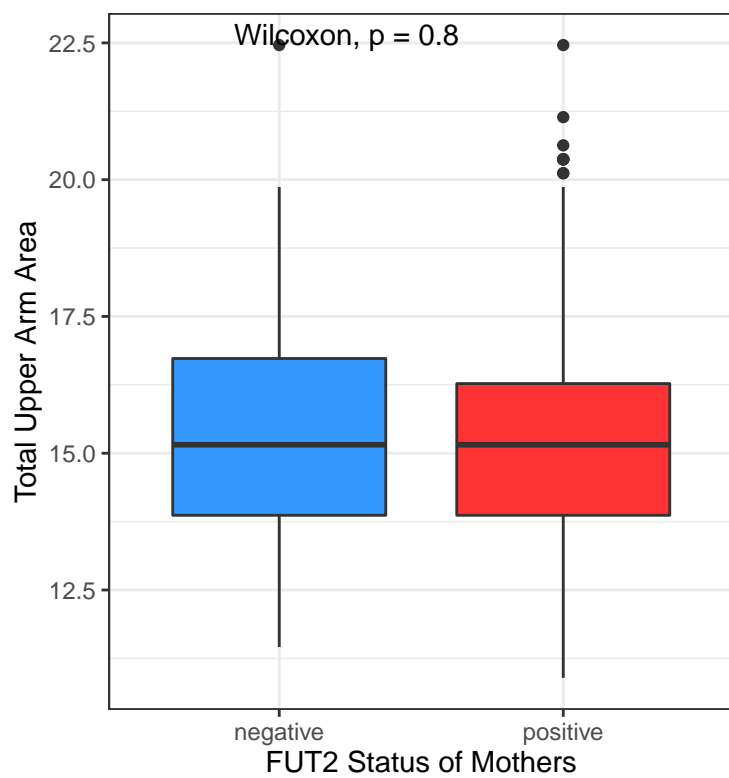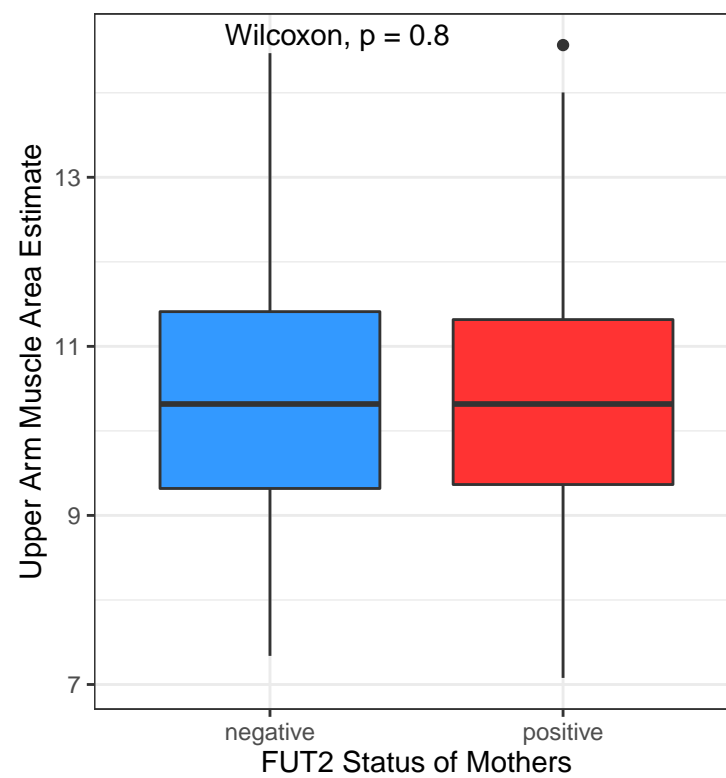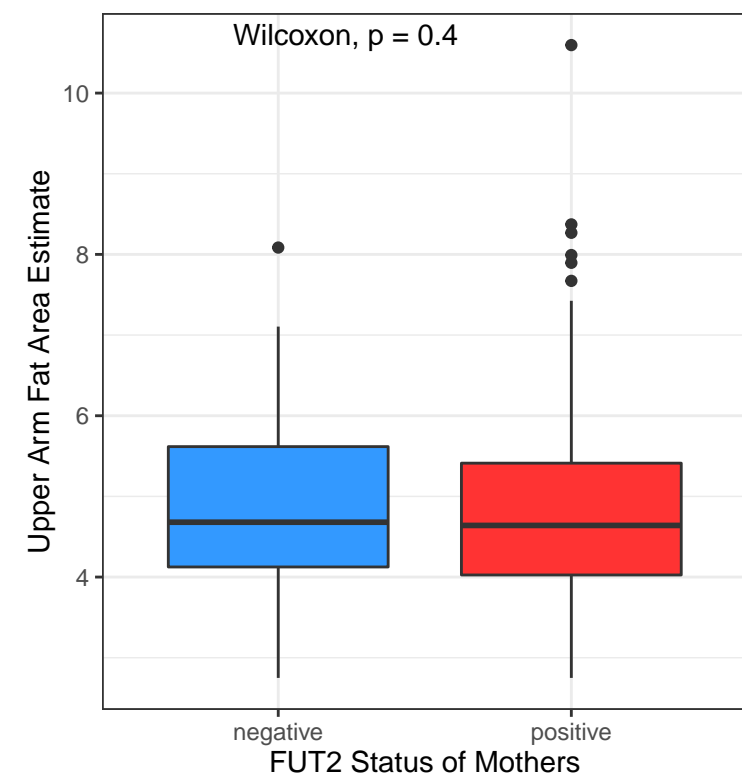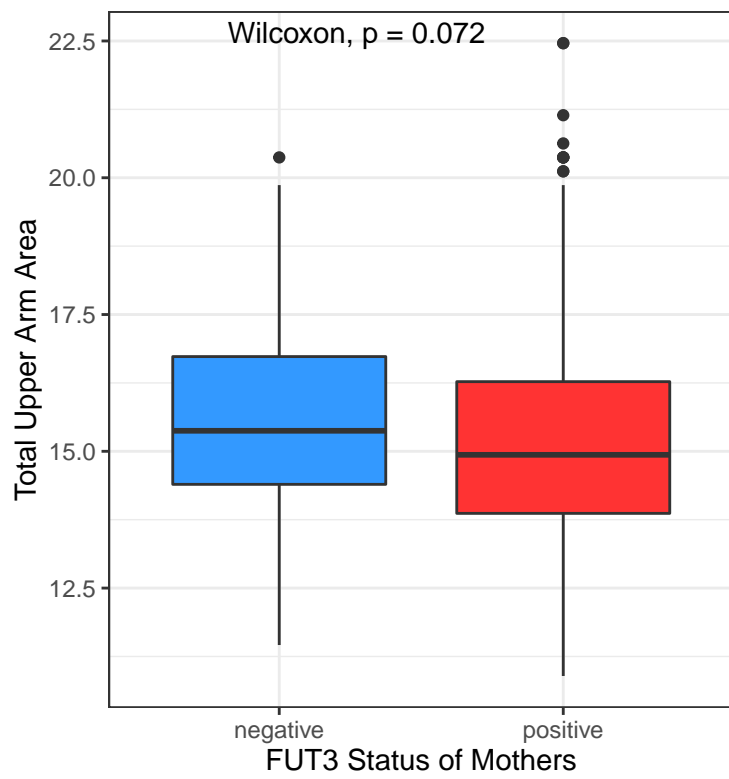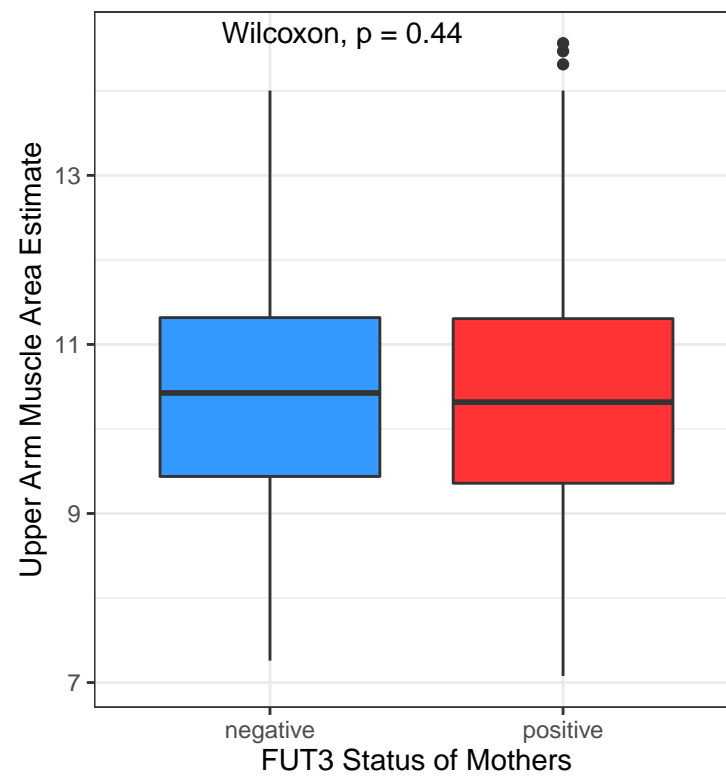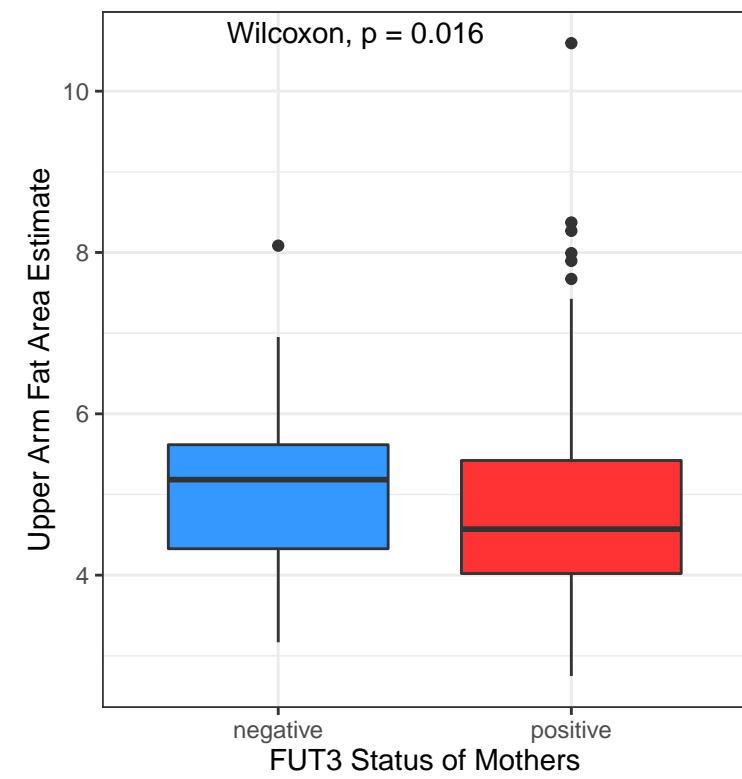

Supplement: Supplementary file 2 — Supplementary Information 2. [file 41598_2022_23616_MOESM2_ESM.pdf]

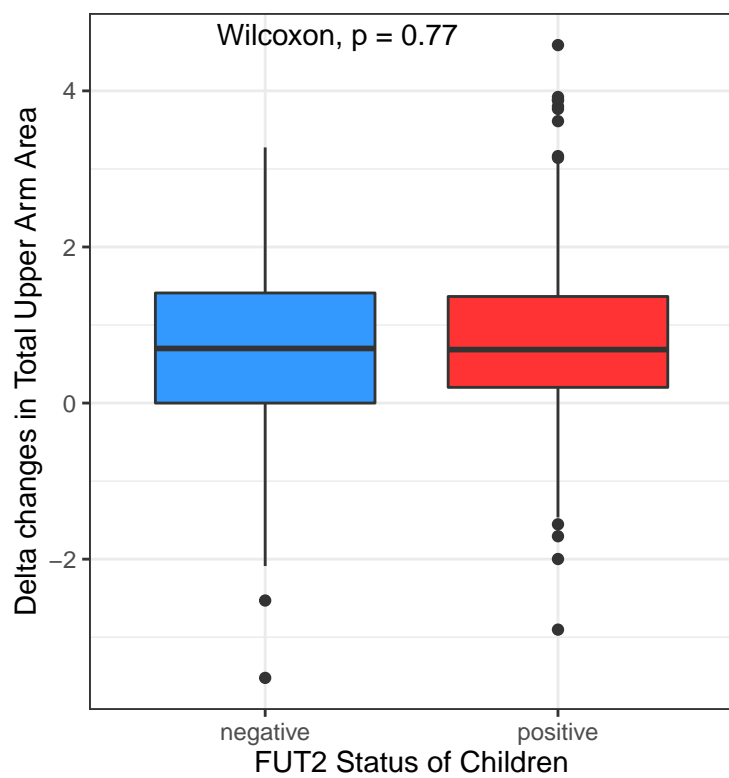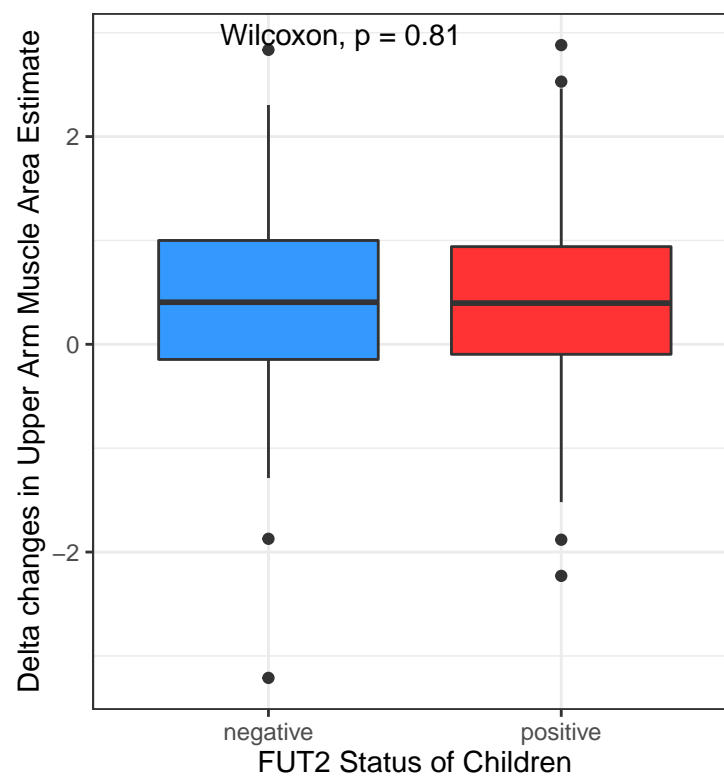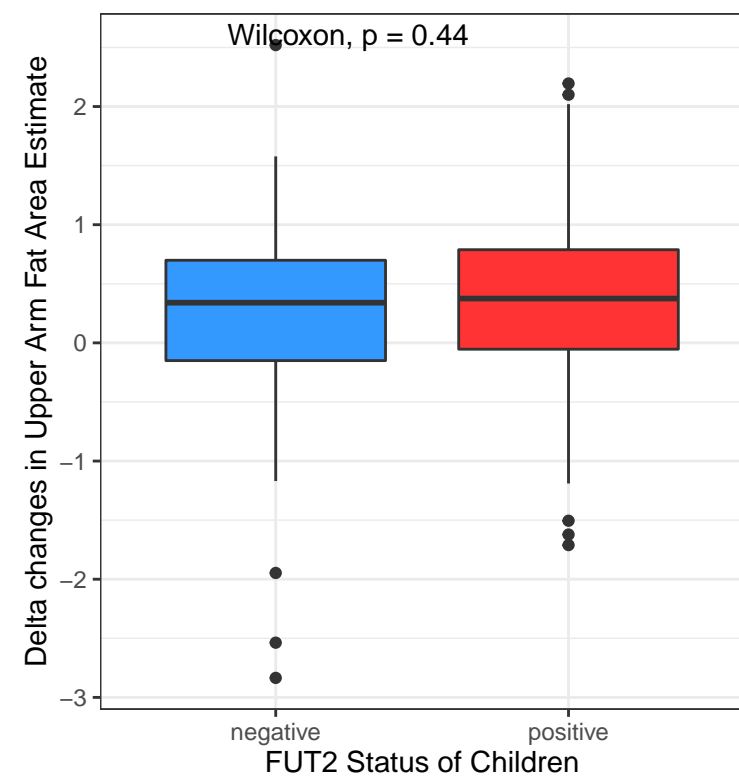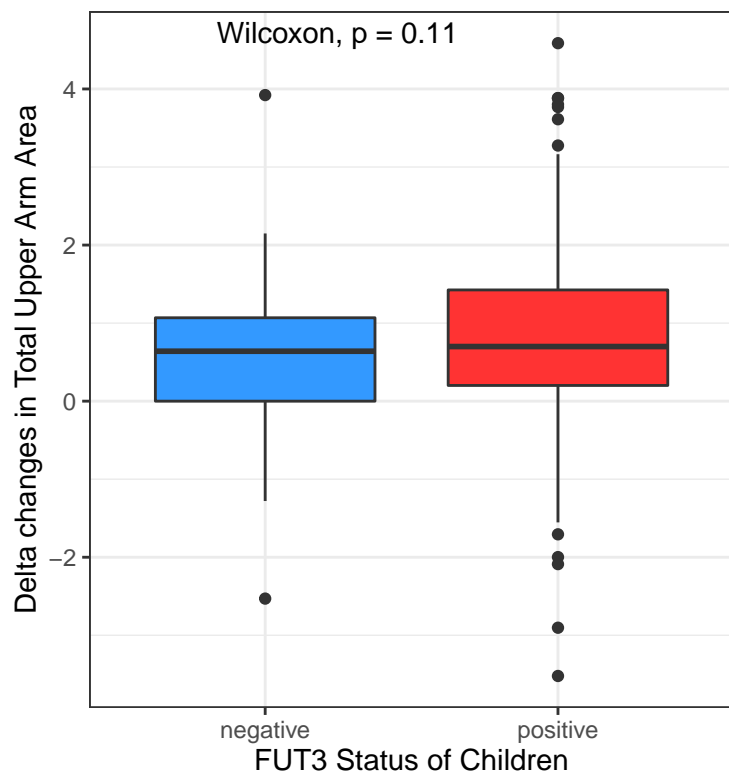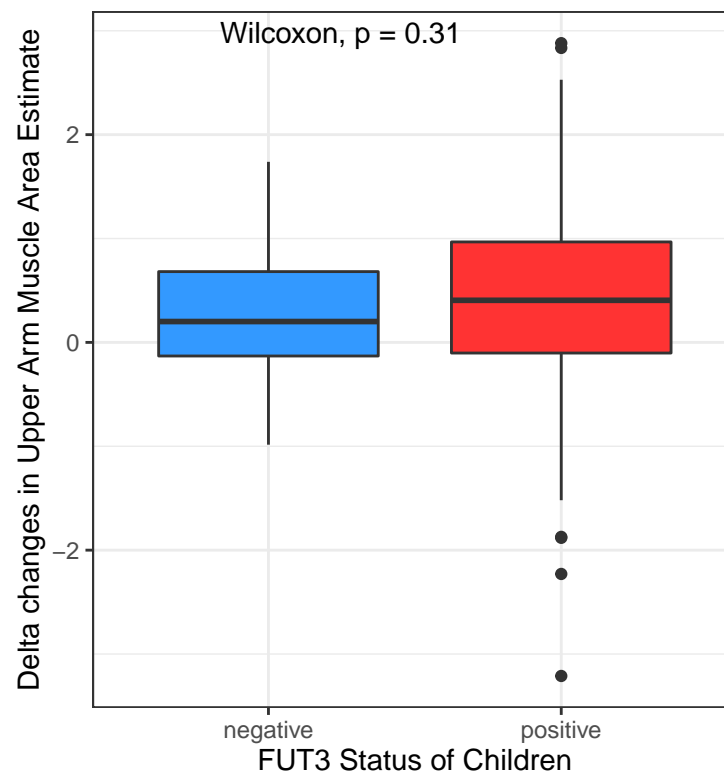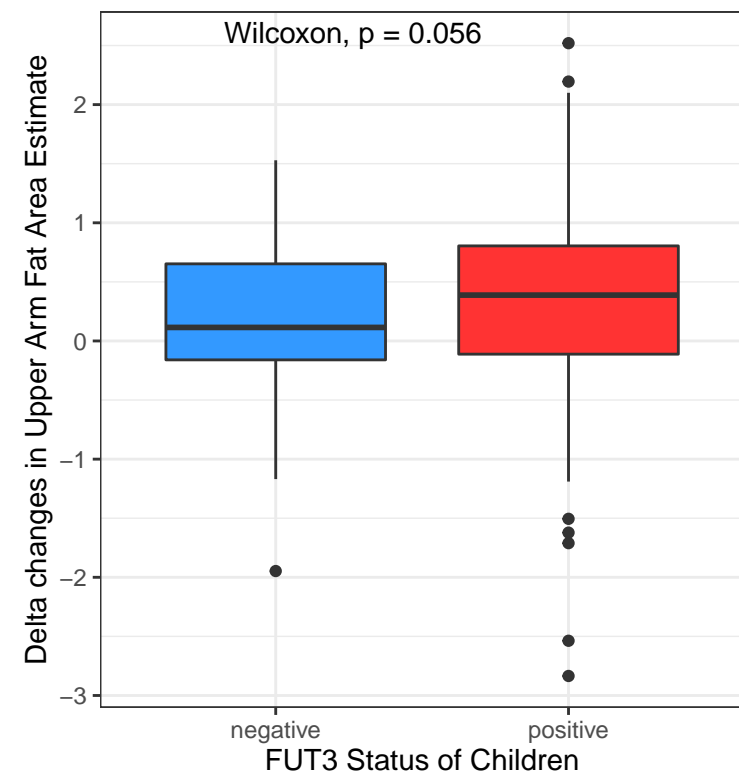

Supplement: Supplementary file 3 — Supplementary Information 3. [file 41598_2022_23616_MOESM3_ESM.pdf]

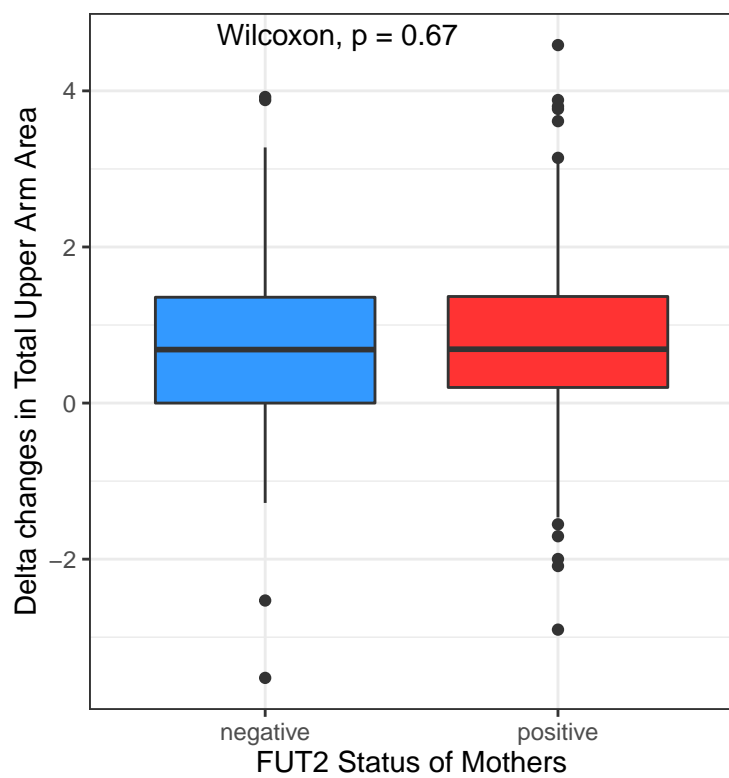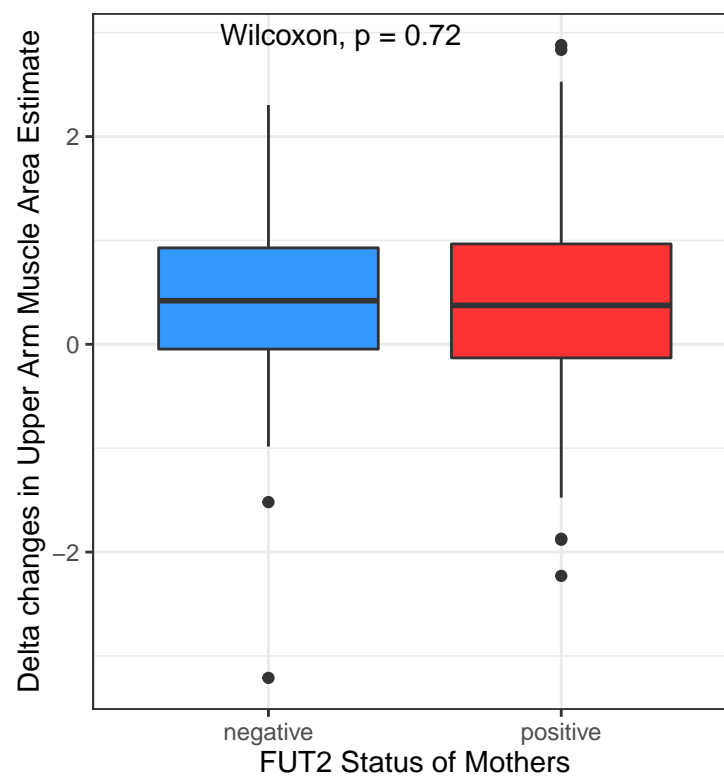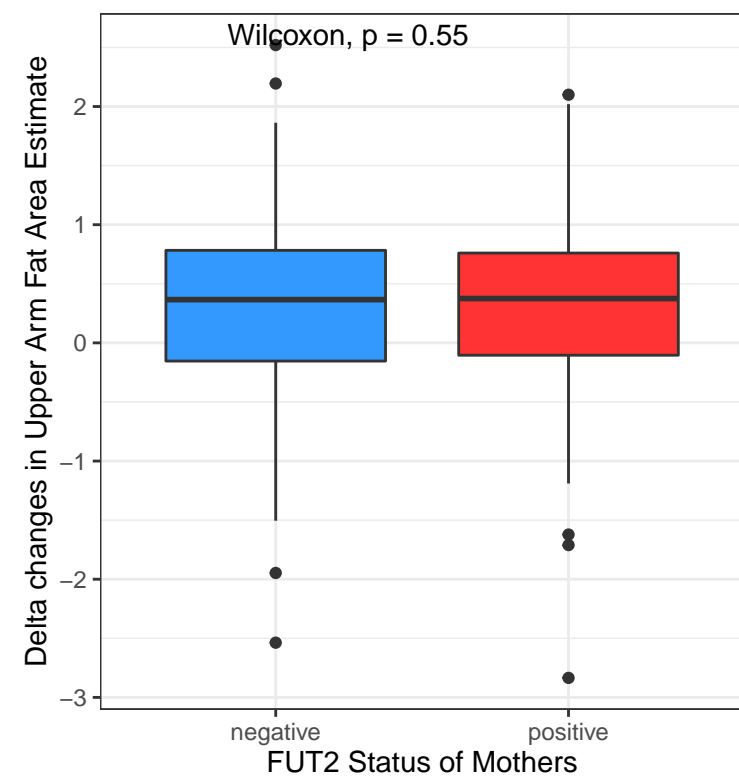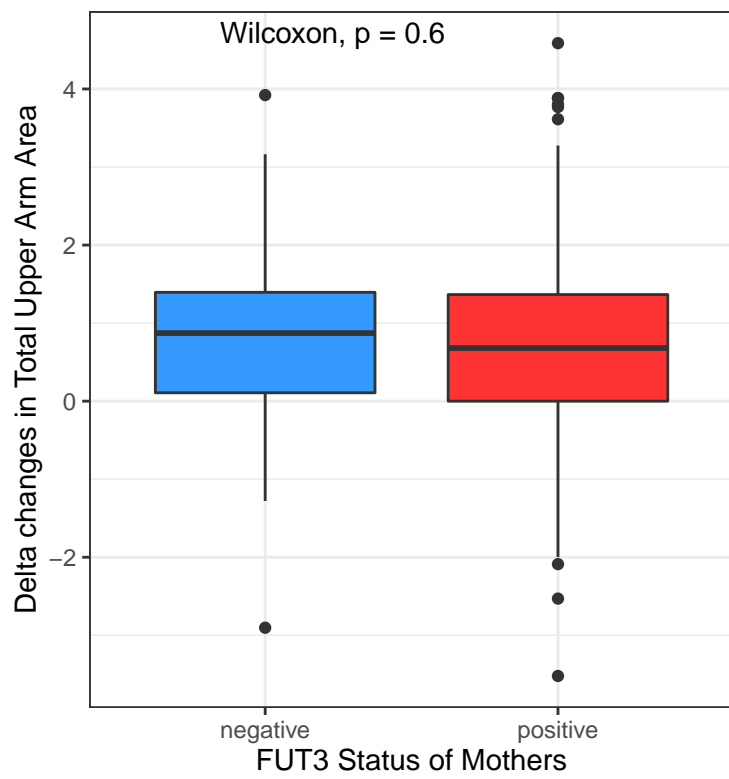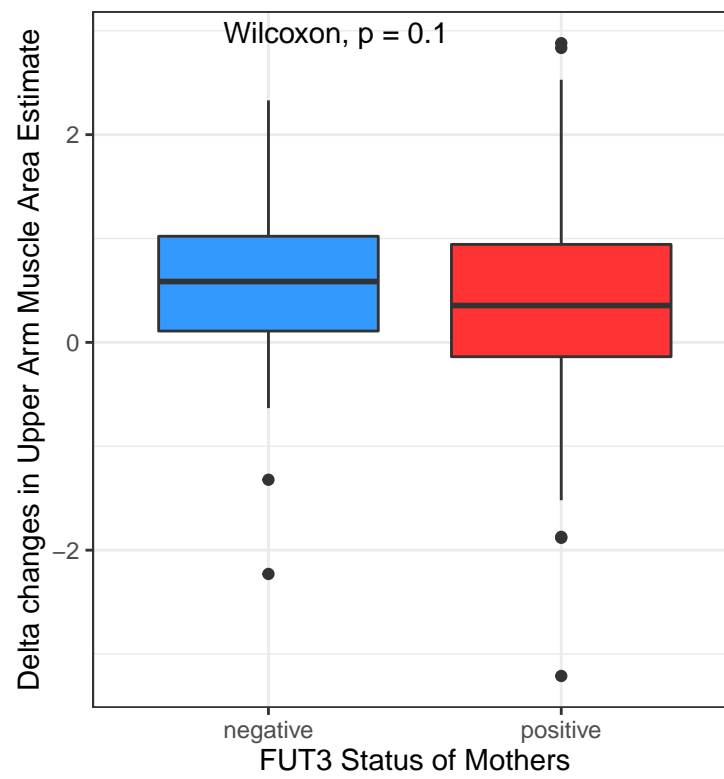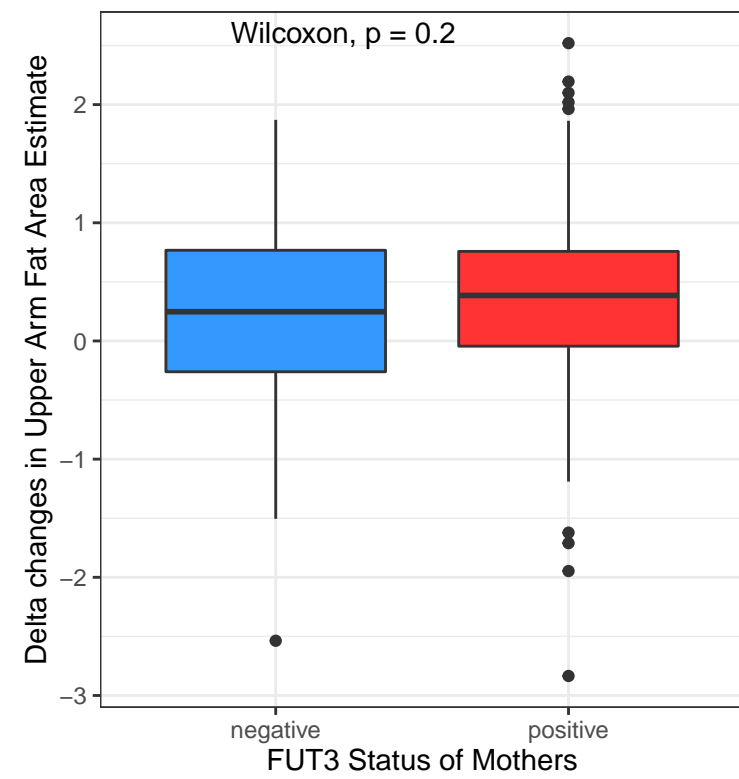

Supplement: Supplementary file 4 — Supplementary Information 4. [file 41598_2022_23616_MOESM4_ESM.pdf]
