# Supplementary material for: Maternal and child FUT2 and FUT3 status demonstrate relationship with gut health, body composition and growth of children in Bangladesh
Source: Sci Rep. 2022 Nov 5;12:18764. doi: 10.1038/s41598-022-23616-9 (PMC9637127; doi:10.1038/s41598-022-23616-9)
Supplement: Supplementary file 5 — Supplementary Information 5. [file 41598_2022_23616_MOESM5_ESM.pdf]

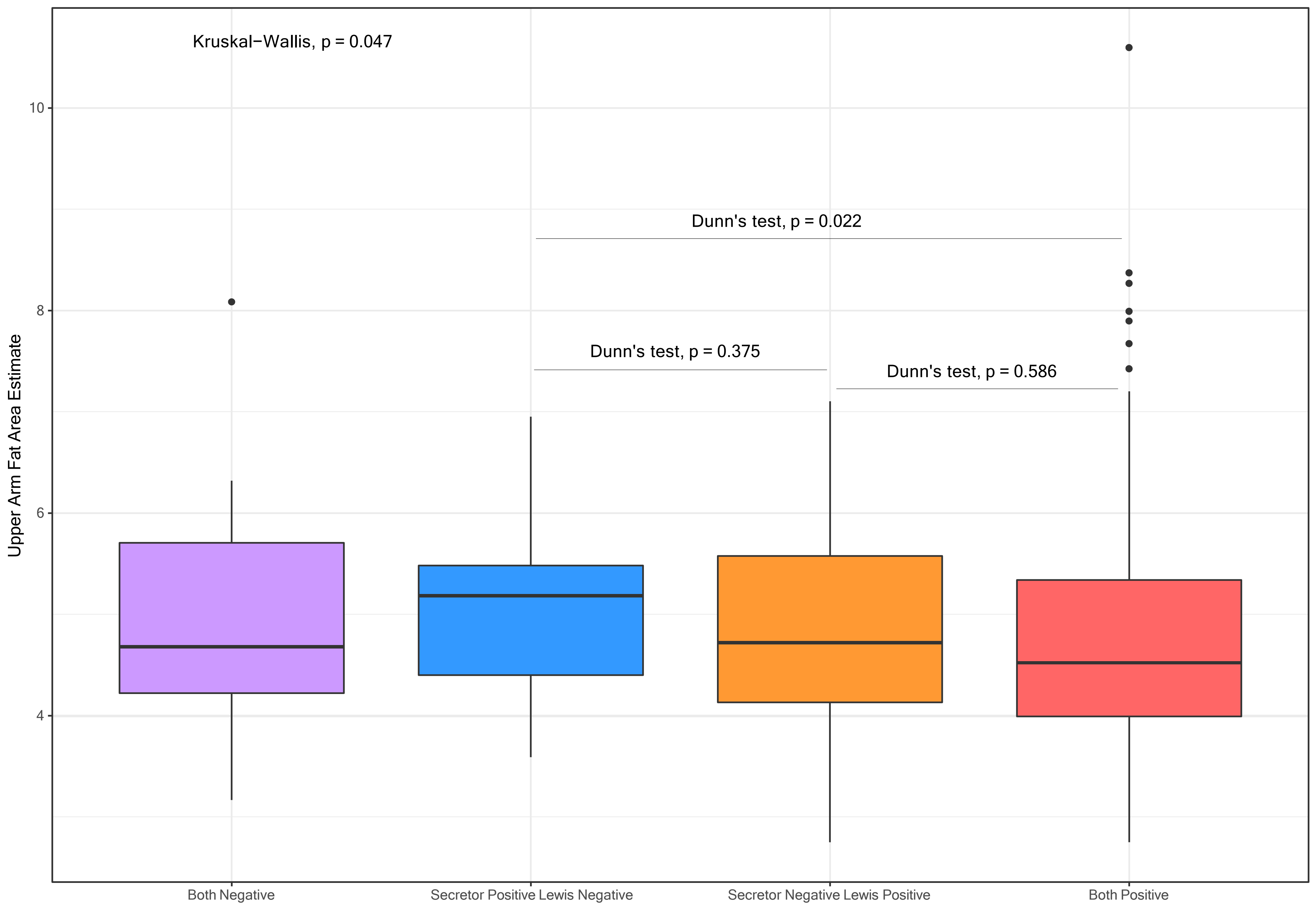
Kruskal−Wallis, p = 0.047
Dunn's test, p = 0.022
Dunn's test, p = 0.375
Dunn's test, p = 0.586
10
8
6
4
Upper Arm Fat Area Estimate
Both Negative
Secretor Positive Lewis Negative
Secretor Negative Lewis Positive
Both Positive
