# Supplementary material for: Maternal and child FUT2 and FUT3 status demonstrate relationship with gut health, body composition and growth of children in Bangladesh
Source: Sci Rep. 2022 Nov 5;12:18764. doi: 10.1038/s41598-022-23616-9 (PMC9637127; doi:10.1038/s41598-022-23616-9)
Supplement: Supplementary file 6 — Supplementary Information 6. [file 41598_2022_23616_MOESM6_ESM.docx]

**Supplementary figure legends:**

**Figure S1:** Body composition and FUT status of children

**Figure S2:** Body composition and FUT status of mothers

**Figure S3:** Changes in body composition and FUT status of children

**Figure S4:** Changes in body composition and FUT status of mothers

**Figure S5:** Body composition and combined FUT status (secretor and Lewis together) of mothers
