# Supplementary material for: Maternal and child FUT2 and FUT3 status demonstrate relationship with gut health, body composition and growth of children in Bangladesh
Source: Sci Rep. 2022 Nov 5;12:18764. doi: 10.1038/s41598-022-23616-9 (PMC9637127; doi:10.1038/s41598-022-23616-9)
Supplement: Supplementary file 7 — Supplementary Information 7. [file 41598_2022_23616_MOESM7_ESM.docx]

**Maternal and Child FUT2 and FUT3 status demonstrate relationship with gut health, body composition and growth of children in Bangladesh**

**Table S1: Changes in growth of children on the basis of FUT status of children**

|  | **Secretor Positive Children (N=408)** | **Secretor Negative Children (N=408)** | **p-value** | **Lewis Positive Children**  **(N=408)** | **Lewis Negative Children**  **(N=408)** | **p-value** |
| --- | --- | --- | --- | --- | --- | --- |
| DLAZ, Median (q1, q3) | 0.02 (-0.14, 0.25) | -0.005 (-0.17, 0.2) | 0.25 | 0.005 (-0.17, 0.23) | 0.09 (-0.10, 0.25) | 0.21 |
| DWAZ, Median (q1, q3) | 0.04 (-0.18, 0.30) | 0.02 (-0.24, 0.28) | 0.49 | 0.05 (-0.19, 0.31) | -0.07 (-0.27, 0.23) | 0.07 |
| DWLZ, Median (q1, q3) | 0.04 (-0.30, 0.38) | 0.04 (-0.31, 0.36) | 0.86 | 0.08 (-0.30, 0.40) | -0.12 (-0.47, 0.20) | 0.02 |

**Table S2: Changes in growth of children on the basis of FUT status of mother**

|  | **Secretor Positive Mothers (N=408)** | **Secretor Negative Mothers (N=408)** | **p-value** | **Lewis Positive Mothers**  **(N=408)** | **Lewis Negative Mothers**  **(N=408)** | **p-value** |
| --- | --- | --- | --- | --- | --- | --- |
| DLAZ, Median (q1, q3) | 0.02 (-0.17, 0.24) | -0.01 (-0.14, 0.21) | 0.37 | 0.01 (-0.16, 0.25) | 0.00 (-0.18, 0.18) | 0.32 |
| DWAZ, Median (q1, q3) | 0.01 (-0.19, 0.28) | 0.10 (-0.21, 0.36) | 0.37 | 0.03 (-0.20, 0.29) | 0.10 (-0.19, 0.32) | 0.85 |
| DWLZ, Median (q1, q3) | 0.03 (-0.31, 0.34) | 0.17 (-0.28, 0.44) | 0.13 | 0.03 (-0.31, 0.37) | 0.17 (-0.23, 0.43) | 0.39 |
