# Supplementary material for: Maternal and child FUT2 and FUT3 status demonstrate relationship with gut health, body composition and growth of children in Bangladesh
Source: Sci Rep. 2022 Nov 5;12:18764. doi: 10.1038/s41598-022-23616-9 (PMC9637127; doi:10.1038/s41598-022-23616-9)
Supplement: Supplementary file 8 — Supplementary Information 8. [file 41598_2022_23616_MOESM8_ESM.docx]

**Table S3: Changes in gut health biomarkers of children on the basis of FUT status of children**

|  | **Secretor Positive Children (N=408)** | **Secretor Negative Children (N=408)** | **p-value** | **Lewis Positive Children**  **(N=408)** | **Lewis Negative Children**  **(N=408)** | **p-value** |
| --- | --- | --- | --- | --- | --- | --- |
| MPO, Median (q1, q3) | 2005 (951.5, 4954) | 2047.2 (659.6, 5433) | 0.99 | 1982 (845.2, 4991.8) | 2829 (1106, 7149) | 0.08 |
| NEO, Median (q1, q3) | 1485 (743, 2868) | 1410 (601.2, 2590.8) | 0.41 | 1457 (707.5, 2731) | 1667.5 (911.5, 2712.8) | 0.38 |
| AAT, Median (q1, q3) | 0.50 (0.22, 0.78) | 0.47 (0.25, 0.77) | 0.70 | 0.48 (0.22, 0.77) | 0.46 (0.25, 0.84) | 0.43 |
| Reg1B | 75.4 (35.4, 96.6) | 58.1 (29.1, 87.7) | 0.032 | 69.9 (32.7, 92.2) | 77.3 (47.7, 96.7) | 0.19 |

**Table S4: Changes in gut health biomarkers of children on the basis of FUT status of mother**

|  | **Secretor Positive Mothers (N=408)** | **Secretor Negative Mothers (N=408)** | **p-value** | **Lewis Positive Mothers**  **(N=408)** | **Lewis Negative Mothers**  **(N=408)** | **p-value** |
| --- | --- | --- | --- | --- | --- | --- |
| MPO, Median (q1, q3) | 2095.0 (907.5, 5023.2) | 1639 (712.5, 5412.5) | 0.43 | 2048.2 (902.2, 5158.8) | 1774 (793, 5030) | 0.46 |
| NEO, Median (q1, q3) | 1452 (764, 2557) | 1506 (600.5, 2982.5) | 0.77 | 1510 (744, 2865) | 1207 (642.5, 2230.5) | 0.21 |
| AAT, Median (q1, q3) | 0.50 (0.23, 0.79) | 0.45 (0.18, 0.74) | 0.49 | 0.48 (0.19, 0.79) | 0.50 (0.31, 0.76) | 0.76 |
| Reg1B | 71.1 (32.5, 92.4) | 70.8 (42.7, 94.7) | 0.61 | 70.6 (34.7, 93.4) | 77.6 (33.6, 93.5) | 0.78 |
